# Different Outcomes of Anti-VEGF Treatment for Neovascular AMD according to Neovascular Sutypes and Baseline Features: 2-Year Real-Life Clinical Outcomes

**DOI:** 10.1155/2021/5516981

**Published:** 2021-05-24

**Authors:** Alessandro Arrigo, Andrea Saladino, Emanuela Aragona, Stefano Mercuri, Ugo Introini, Francesco Bandello, Maurizio Battaglia Parodi

**Affiliations:** Department of Ophthalmology, IRCCS San Raffaele Scientific Institute, Via Olgettina 60, 20132 Milan, Italy

## Abstract

**Purpose:**

To evaluate the effects of anti-VEGF treatment of neovascular age-related macular degeneration (nAMD) in a real-life clinical setting.

**Methods:**

Study design is a retrospective case series. Naïve nAMD patients treated with intravitreal injection of aflibercept or ranibizumab were analyzed over a 24-month follow-up. Each patient received the loading dose, followed by a PRN regimen. Patients were further subdivided into subgroups according to macular neovascularization type, best corrected visual acuity (BCVA) at baseline (BCVA > 0.3 LogMAR and BCVA ≤ 0.3 LogMAR), and different anti-VEGF drugs. Primary outcome was the changes in BCVA and central macular thickness (CMT) over 24 months. Secondary outcomes included the influence of the selected drug and of the baseline BCVA on the final outcomes.

**Results:**

439 patients (224 males; 51%) with naïve AMD-related macular neovascularization were included in the analyses. Mean age was 78 ± 8 years old. Compared to baseline evaluations, not significant BCVA changes were found at 1-year and 2-year examinations. CMT was significantly reduced at both 1-year and 2-year follow-ups (*p* < 0.01). Classic, polypoidal choroidal vasculopathy and mixed subtypes significantly correlated with worse visual outcome (*p* < 0.01). Overall, baseline BCVA significantly correlated with both 1-year and 2-year follow-up changes (*p* < 0.01). Moreover, BCVA at 1-year significantly correlated with BCVA changes at 2-year follow-up (*p* < 0.01). Furthermore, CMT changes from baseline significantly correlated with both 1-year and 2-year follow-up measurements (*p* < 0.01).

**Conclusion:**

Anti-VEGF approach is generally effective in stopping nAMD progression in our real-life analysis. No difference was found comparing patients treated with ranibizumab and aflibercept, nor in patients with drug switching.

## 1. Introduction

Age-related macular degeneration (AMD) is a leading cause of vision loss which affects people older than 50 years old in developed countries. The European prevalence in 2040 is estimated to reach 21 million of people affected by the early stage and the 4.8 million by the late one [1].Advanced stages could be divided into two groups: geographic atrophy (GA) and neovascular or wetform. The neovascular form, which is the most frequent, is characterized by a severe vision loss caused by the growth of neovessels under or within the macula [2]. Even if the pathogenesis is still not completely understood, vascular endothelial growth factor (VEGF) is surely involved in the neovascularization promotion [3–6]. Neovascular form rapidly results in central vision loss but with the introduction of Anti-VEGF drugs, and it became possible to stop the natural evolution of nAMD. Ranibizumab was approved by the US and European Union, respectively, in 2006 and 2007 following the phase III clinical trials MARINA and ANCHOR, while aflibercept was approved in 2011-2012 after showing noninferiority to ranibizumab with a lower number of injections by VIEW 1 and 2 studies. In a real-life setting, missed appointments elongate intervals between injections and comorbidities. The stringent inclusion and exclusion criteria, relatively small patient numbers, intensive treatment regimens, and limited duration of these clinical trials may not necessarily reflect real-world experience. For these reasons, it is fundamental to evaluate outcomes in an everyday scenario in order to discover weaknesses in the routine and find out how to resolve them. This difference leads to the need to analyze the results we achieve with our patients in a daily “real life” scenario. Even if a large number of systematic reviews have proven the practical limitation of anti-VEGF treatment in clinical routinely uses [7–9], most of the studies showed not as pronounced anatomical and functional gains as in the clinical trial setting [7, 10–13]. Aim of the present study is to analyze the clinical outcomes of anti-VEGF treatment in a tertiary referral centre over a 2-year follow-up.

## 2. Materials and Methods

The study was designed as a retrospective investigation to describe the real-life management in patient affected by nAMD treated with ranibizumab or aflibercept over a 24-month follow-up. Switching to a different anti-VEGFdrug was allowed at ophthalmologist's discretion on the basis of predefined criteria. Study protocol was approved by the Institutional Review Board of Scientific Institute San Raffaele, and the procedures followed the straits of the Declaration of Helsinki. Written informed consent was acquired from all the patients included in the present study. Patient's recruitment started from January 2014 and ended in December 2016 in order to permit the follow-up analysis.

Naive neovascular AMD was diagnosed on the basis of clinical data and multimodal imaging. Patients underwent a loading dose of 3 injections, followed by a PRN treatment regimen with monthly examination. Retreatments were administered when reactivation or progression of the disease was registered, on the basis of the identification of retinal or subretinal fluid on optical coherence tomography (OCT), leakage on fluorescein angiography/indocyanine green angiography, or new macular hemorrhages.

Neovascular AMD was diagnosed through dilated fundoscopy examination, by OCT, and fluorescein angiography. Patients underwent monthly ophthalmological examinations, including measurement of best corrected visual acuity (BCVA), using standard ETDRS charts, funduscopicexamination, Goldman applanation tonometry, and OCT (Spectralis HRA + OCT; Heidelberg Engineering, Heidelberg, Germany). The acquisition protocol used a 19-line raster SD-OCT scheme, with each line at 240 *μ*m intervals, covering an area of 20 × 15 degrees (approximately 6 × 4.5 mm^2^).

Any other retinal disorders or any condition (systemic diseases or treatment) which could interfere with the anti-VEGF therapy or the clinical outcomes was considered as exclusion criteria.

The criteria followed to the treatment switching included CMT reduction < 30% and/or BCVA deterioration > 10 ETDRS letters.

Primary outcomes were the change in BCVA and central macular thickness (CMT) over the 24-month follow-up. Secondary outcomes included the influence of the selected drug and of the baseline BCVA on the final outcomes.

Statistical analysis was achieved by both Microsoft Excel 2013 (Microsoft Corporation, Washington, USA) and SPSS Statistics Version 23.0 (IBM, Armonk, NY). Each result is expressed as mean ± SD (standard deviation), and a *p* value <0.05 is estimated as statistically significant. Comparison between parameters at baseline and follow-ups were expressed by means of coupled *t*-test.

In order to analyze subgroups of patients, the analysis of variance (ANOVA) followed by the Tukey HSD test for posthoc analysis was used.

Correlation analysis between numeric variables was obtained and analyzed with Tau-Kendall correlation coefficient.

## 3. Results

Overall, 479 naïve AMD-related macular neovascularization (MNV) patients were recruited. Twenty patients were excluded due to high media opacity; another 20 patients were excluded because of uncontrolled arterial hypertension (12 patients) and glaucoma (8 patients). The mean age was 78 ± 8 years old; 51% were male, and the 49% were female (*p* > 0.05) that were evaluated. The entire cohort was subdivided in different groups using a BCVA cut-off (0.3 LogMAR) and MNV types. Percentages for each CNV type were 56.8% for type 1 (occult form), 26.1% for the classic form (type 2), 13.5% for mixed form, and finally, 3.6% for polypoidal choroidal vasculopathy. Ranibizumab or aflibercept monotherapy was used in most of the cases over the follow-up, whereas a shifting was registered in 28% of cases that was also made.

All clinical data are listed in Table 1.

Compared to baseline evaluations, not significant BCVA changes were achieved at 1-year and 2-year follow-up (all *p* > 0.05). On the other hand, CMT was significantly reduced at both 1-year and 2-year follow-ups (all *p* < 0.01) (Table 2).

Classic, polypoidal choroidal vasculopathy and mixed MNV subtypes significantly correlated with worse visual outcome (*p* < 0.01) (Table 2).

Overall, baseline BCVA significantly correlated with final BCVA at 1-year and 2-year follow-ups (*p* < 0.01) (Table 2). In particular, eyes characterized by baseline BCVA < 0.3 LogMAR did not show significant improvements (Table 3). Furthermore, CMT changes from baseline significantly correlated with both 1-year and 2-year follow-up measurements (*p* < 0.01) (Table 2).

Eyes with worse baseline BCVA (>0.3 LogMAR) were characterized by greater mean CMT (*p* < 0.01) compared to patients with better baseline BCVA (≤0.3 LogMAR), at both baseline (416 ± 136 vs 343 ± 83 *μ*m) and 1-year (359 ± 105 vs 308 ± 71 *μ*m) measurements (Table 3). Patients that started with better visual acuity have higher quality BCVA at the end of the study.

The specific analyses regarding the effect of the drug, with ranibizumab or aflibercept monotherapy, as well as patients that underwent a switch to the other drug during the treatment period, revealed that no significant difference was found; although, patients shifting to the other drug received a significantly higher number of intravitreal injections (*p* < 0.01), without remarkable functional and anatomical changes (Table 4).

## 4. Discussion

Our study described the achieved outcome of nAMD patients treated by anti-VEGF injections in real-life. Despite the positive results, application of clinical trial strategy in the real-world practice is hardly achievable because of many reasons, especially regarding the strict inclusion and exclusion criteria along with the patients' compliance. In real-life setting, missed appointments, which elongate intervals between injections and also comorbidities, which discourage to attend visits, are frequent [13]. For these reasons, it is essential to evaluate the clinical outcomes in an everyday scenario in order to identify all the weaknesses and find out how to resolve them. The present retrospective analysis is based on a cohort of 439 AMD patients, treated with aflibercept, ranibizumab, or both over a 24-month follow-up. Considering the entire cohort, no statistically significant difference was found in BCVA values between baseline, first year, and second year BCVA. Subdividing the patients into two subcategories on the baseline BCVA using the cut-off of 0.3 LogMAR, we registered an inverse relation between VA and CMT. The patients with a higher central macular thickness had a lower LogMAR VA. However, a statistically significant CMT reduction was found from baseline to the final examination. Interestingly, although the subgroup with VA > 0.3 LogMAR and the other subgroup with VA < 0.3 LogMAR started with different mean CMT (416 *μ*m vs 342 *μ*m, respectively), at the end of the follow-up, their CMT turned out to be similar (314 *μ*m vs 300 *μ*m). This may be explained as the consequence of adopting CMT as a clinical parameter to guide the retreatment planning.

Our results confirm that the anti-VEGF approach in the real-life practice can lead to a stabilization of the visual function, stopping the natural progression towards a visual loss. In particular, our data are in line with those of the COMPASS study [14].On the other hand, some studies included patients with lower baseline BCVA, who were more prone to gain vision [7, 8, 15].

Almost one third of our patients were shifted from one drug to another one on the basis of the unsatisfactory response at the ophthalmologist's discretion. Nevertheless, no improvement in BCVA and CMT was registered in the patients shifted to the new agent, even though the global number of injections increased because most of the ophthalmologist decided to restart with a loading phase on the new drug. Contrasting results have been obtained by other studies [16–18], but in our experience, switching is not explicitly recommendable, because it is potentially more expensive for the healthcare system (increasing the global number of injections) and devoid of clear evidence of positive effects.

MNV type stratification found that, differently from classic, polypoidal choroidal vasculopathy and mixed pattern, occult MNV (36% of the cohort) showed better outcomes comparing baseline to year 1, baseline to year 2, and even year 1 to year 2.

Overall, our retrospective analysis shows a clinical outcome slightly inferior to those revealed by ANCHOR and MARINA trials [19, 20].The reasons why routine clinical settings do not improve VA like clinical trials could be explained by less strict monitoring of patients.

More specifically, the most important causes included the distance of the patient from the hospital, comorbidities that can discourage patients to be treated, and long waiting lists, which elongated the intervals among injections. In addition, we need to underline that the retrospective design of the study is an inherent bias per se. Moreover, we are aware that the adopted PRN treatment regimen may have an impact on the outcome achieved, since PRN methodology is known to yield inferior outcome, compared with fixed or treat and extend regimens. Moreover, we are aware that the lack of extremely positive outcome may be explained considering that real-life setting is quite different from sponsored studies.

Further studies on national basis are warranted to implement the real-life use of the anti-VEGF approach for the management of AMD.

## Figures and Tables

**(a):** 

Clinical data									
	BCVA baseline	BCVA 1 y	BCVA 2 y	CMT baseline	CMT 1 y	CMT 2 y	N. IV 1 y	N. IV 2 y	TOT N. IV
Mean	0.46	0.40	0.42	380	334	307	6.1	3.5	9.6
STD	0.33	0.37	0.38	118	93	62	1.7	2.1	3.8

**(b):** 

Clinical data		
*p* value	BCVA	CMT
Baseline vs 1 y	0.001	0.001
Baseline vs 2 y	0.01	0.001
1 y vs 2 y	0.04	0.01

**Table 2:** Correlation analysis. The following abbreviations are used: MNV: macular neovascularization; BCVA: best-corrected visual acuity; CMT central macular thickness.

Correlation analysis			
		Tau-Kendall value	*p* value
MNV Type	BCVA 1 y	0.349	<0.01
	BCVA 2 y	0.329	<0.01
BCVA baseline	BCVA 1 y	0.55	<0.01
	BCVA 2 y	0.55	<0.01
	CMT baseline	0.259	<0.01
BCVA 1 y	BCVA 2 y	0.709	<0.01
CMT baseline	CMT 1 y	0.254	<0.01
CMT 1 y	CMT 2 y	0.317	<0.01

**(a):** 

Stratified analysis on the basis of BCVA										
		BCVA baseline	BCVA 1 y	BCVA 2 y	CMT baseline	CMT 1 y	CMT 2 y	N. IV 1 y	N. IV 2 y	TOT N. IV
BCVA ≤ 0.3	MEAN	0.178	0.181	0.2	342	308	300	6.2	3.7	9.9
	STD	0.097	0.162	0.191	83	71	65	1.6	2.2	3.2
BCVA > 0.3	MEAN	0.701	0.595	0.61	416	358	314	6.0	3.3	9.3
	STD	0.279	0.403	0.408	136	105	59	1.7	2	3.3

**(b):** 

Stratified analysis on the basis of BCVA			
*p* value		BCVA	CMT
BCVA ≤ 0.3	Baseline vs 1 y	>0.05	<0.01
		<0.01	<0.01
	Baseline vs 2 y	>0.05	<0.01
		<0.01	<0.01
	1 y vs 2 y	>0.05	0.03
		>0.05	<0.01

**Table 4:** Stratified analysis on the basis of anti-VEGF treatment. The following abbreviations are used: BCVA: best-corrected visual acuity; CMT: central macular thickness.

Stratified analysis on the basis of anti-VEGF treatment										
		BCVA baseline	BCVA 1 y	BCVA 2 y	CMT baseline	CMT 1 y	CMT 2 y	N. IV 1 y	N. IV 2 y	TOT N. IV
Ranibizumab	MMean	0.47	0.41	0.45	364	329	307	5.5	2.9	8.4
	STD	0.36	0.4	0.42	125	84	63	1.7	1.8	3.2
Aflibercept	MMean	0.504	0.45	0.44	402	345	299	5.3	2.6	7.9
	STD	0.34	0.4	0.39	136	105	62	1.4	1.8	2.8
Switch	Mean	0.384	0.33	0.34	373	326	317	7.6	4.9	12.5
	STD	0.287	0.28	0.3	76	88	61	1.6	2.0	3.6
